# The Role of Maternal Nutrition on Oocyte Size and Quality, with Respect to Early Larval Development in The Coral-Eating Starfish, *Acanthaster planci*

**DOI:** 10.1371/journal.pone.0158007

**Published:** 2016-06-21

**Authors:** Ciemon Frank Caballes, Morgan S. Pratchett, Alexander M. Kerr, Jairo A. Rivera-Posada

**Affiliations:** 1 ARC Centre of Excellence in Coral Reef Studies, James Cook University, Townsville, Queensland, Australia; 2 University of Guam–Marine Laboratory, UOG Station, Mangilao, Guam, United States of America; 3 Environmental Corporation, University of Antioquia, Medellin, Colombia; Michigan State University, UNITED STATES

## Abstract

Variation in local environmental conditions can have pronounced effects on the population structure and dynamics of marine organisms. Previous studies on crown-of-thorns starfish, *Acanthaster planci*, have primarily focused on effects of water quality and nutrient availability on larval growth and survival, while the role of maternal nutrition on reproduction and larval development has been overlooked. To examine the effects of maternal nutrition on oocyte size and early larval development in *A*. *planci*, we pre-conditioned females for 60 days on alternative diets of preferred coral prey (*Acropora abrotanoides*) versus non-preferred coral prey (*Porites rus*) and compared resulting gametes and progeny to those produced by females that were starved over the same period. Females fed *ad libitum* with *Acropora* increased in weight, produced heavier gonads and produced larger oocytes compared to *Porites*-fed and starved females. Fed starfish (regardless of whether it was *Acropora* or *Porites*) produced bigger larvae with larger stomachs and had a higher frequency of normal larvae that reached the late bipinnaria / early brachiolaria stage compared to starved starfish. Females on *Acropora* diet also produced a higher proportion of larvae that progressed to more advanced stages faster compared to *Porites*-fed starfish, which progressed faster than starved starfish. These results suggest that maternal provisioning can have important consequences for the quality and quantity of progeny. Because food quality (coral community structure) and quantity (coral abundance) varies widely among reef locations and habitats, local variation in maternal nutrition of *A*. *planci* is likely to moderate reproductive success and may explain temporal and spatial fluctuations in abundance of this species.

## Introduction

Episodic population outbreaks of the crown-of-thorns starfish, *Acanthaster planci*, have resulted in widespread degradation of Indo-Pacific coral reefs [1]. While the ultimate cause of outbreaks is still the subject of debate, most researchers agree that exploring the reproductive biology and early life history of *A*. *planci* is essential in understanding mechanisms that lead to outbreaks. The two most prominent hypotheses that seek to explain the cause(s) of outbreaks, the ‘terrestrial runoff hypothesis’ [2–5] and ‘predator removal hypothesis’ [6,7] are built upon variations in larval survival, growth, and development in response to starvation and predation. Populations of *A*. *planci* are predisposed to major fluctuations due to inherent properties of their life history such as high fecundity [8,9], high fertilization rates [10,11], and short generation times [12,13]. Small environmental and biological changes, therefore, could potentially lead to rapid increases in the abundance of *A*. *planci* [14].

Fluctuations in larval survival and development can have pronounced effects on recruitment rates and hence the dynamics of adult populations [5,15]. Increased larval nutrition has a positive effect on the condition of the larvae of *A*. *planci*, as it does on other marine organisms with planktotrophic larvae. [3]. Past studies on the survival and development of larvae of *A*. *planci* have primarily been centered on the direct effects of nutrient concentrations and exogenous food availability in the water column [3,16–18]. However, the role of maternal nutrition on reproduction and larval development of *A*. *planci* has generally been overlooked. The few studies that have explored effects of maternal condition in *A*. *planci* examined the effects of food availability on gametogenesis. Cheney [19] reported that total deprivation of coral food for one month by caging resulted in shrinking of gonads, deterioration of pyloric caeca, and decrease in total diameter. Conversely, gravid *A*. *planci* collected from Okinawa and starved for 90 days showed no change in the size and condition of gonads even though pyloric caeca were reduced to thin ribbons [20].

The quantity and quality of food available to adult starfish can have flow-on effects on overall reproductive capacity [21]. Coral composition and abundance are often variable in nature [22,23] and local conditions can influence the nutritional status of corallivores, like *A*. *planci*. It is well established that *A*. *planci* have distinct feeding preferences and *Acropora* (along with *Montipora*) are among the most preferred genera, consistently eaten in preference to other locally abundant corals [1,24,25]. Although *Porites* are much less preferred, they are not totally avoided and are often consumed when more preferred species have been depleted [26]. Starved individuals have also been observed towards the end of outbreak events when live coral prey becomes scarce [27].

Natural variation in food availability and nutrient assimilation can influence gamete production in echinoderms [28]. Starvation has been found to result in the failure of gonads to achieve normal size increments [29]. In asteroids, most nutrients used in gametogenesis are processed and stored in the pyloric caecum and delivered directly to the gonads [30]. Echinoderms found in favorable habitats with abundant supply of preferred food items often respond by increasing body weight, gonad size, and pyloric caeca index [21]. For example, Scheibling and Lawrence [31] found that starfish (*Echinaster* sp.) found on seawalls with abundant supply of oysters, sponges, ascidians, and bryozoans were almost three times heavier than individuals from less favorable sites. Moreover, green sea urchins (*Strongylocentrotus droebachiensis*) from shallower depths, where preferred macroalgal food was more abundant, had larger gonads compared to females from food-limited deeper sites [32]. The weight of the pyloric caeca for *Pisaster ochraceus* from wave-exposed sites, where food items are more abundant, was significantly higher compared to starfish from wave-protected sites [33]. Laboratory studies also confirmed that adults on rich food diets during active gametogenesis have higher body weight, gonad size, and pyloric caeca index [21]. This was demonstrated by experimental manipulation of diet in the New Zealand starfish, *Sclarasterias mollis*, where body weight, and gonad and pyloric caeca index increased significantly compared to starved starfish [34,35]. The lipid content of fed starfish was also higher than in starved groups, although protein and carbohydrate contents in gonads did not vary significantly between feeding treatments [35].

Maternal nutrition can alter resource allocation mechanisms and affect nutrient investment in oocytes [36,37]. Oocyte size, fecundity, and oocyte quality can vary with the nutritional history of adults [38–41]. Because oocyte size is a function of maternal investment, selection on oocyte size is a function of maternal fitness [42]. Echinoderms found at sites with abundant food supply and in high food laboratory treatments mostly produced higher numbers of large, high quality oocytes [21]. Female starfish (*Leptasterias epichlora*) from high food availability, exposed sites produced higher numbers of larger oocytes with higher protein content compared to those from sheltered sites [43]. Starfish collected from less favorable sheltered sites subsequently placed under high food ration treatments in the laboratory had more oocytes with larger diameter compared to starfish in low food treatments [43].

For free-spawning invertebrates, the entire maternal contribution to subsequent generations is provided in the oocyte [44]. Following fertilization and gastrulation, the digestive tract differentiates (early bipinnaria stage) and larvae enter the facultative feeding period (FFP); at this stage, larvae are able to feed but do not necessarily require food because maternal provisions from the oocyte are still available [45]. During the initial larval stages, maternal provisioning can have important consequences for starvation resistance, mortality risk from predation, and developmental rates [46,47], ultimately leading to reduced planktonic duration and increased settlement success. In the absence of exogenous food, larvae from large oocytes (high maternal investment) have significant buffering from longer periods of starvation during the FFP [45]. Predation rates during the vulnerable stage of larval development are also moderated by rapid larval development [48].

In this study, we examined the role of experimental variation in maternal nutrition (comparing between individuals that were starved, fed on preferred corals and fed on generally non-preferred coral prey) on the larval growth and early development prior to exogenous feeding by larvae. The effect of maternal nutrition on the following aspects of reproduction and larval development in *A*. *planci* are specifically addressed in this study: (1) adult female morphometrics before and after treatment; (2) gonad and pyloric caeca indexes; (3) oocyte size and shape; (4) fertilization rates; (5) early larval growth; (6) larval survival; and (7) larval development. Few studies have investigated the effect of the nutritional state of the adult *A*. *planci* on oocyte size, fertilization, larval growth and development. This has important implications on the survival of larvae when exogenous food supply is low or absent. Development and growth rates of the larvae of *A*. *planci* are predicted to be low in the absence of enhanced phytoplankton levels [3–5,18]. However, it is not known whether increased maternal investment allows larvae to withstand prolonged periods of starvation and proceed with normal development. Because food quality (coral community structure) and quantity (coral abundance) varies widely between adult populations of *A*. *planci* in coral reefs, any effect of maternal nutrition on larval quality and survivorship may influence the overall reproductive success of *A*. *planci* and help explain marked fluctuations in abundance.

## Methods

### Specimen Collection and Ethics Statement

All experiments were conducted at the University of Guam Marine Laboratory (UOGML) in accordance with regulations set out by the University of Guam and James Cook University. Crown-of-thorns starfish were collected on SCUBA from reefs at the southern end of Ague Point (13.565360°N, 144.819119°E) on the northwest coast of Guam and immediately transported to UOGML. No permits were required to collect *A*. *planci*. Sex was determined by examining contents drawn from gonads along the arm junction using a syringe with a large-bore biopsy needle [13]. Male and female *A*. *planci* were maintained in separate flow-through tanks. Male *A*. *planci* were maintained on a mix of *Acropora* spp., *Porites* spp., and *Pocillopora* spp. corals as soon as they were introduced to holding tanks, while female *A*. *planci* were starved for 10 days prior to being assigned to one of three different feeding treatments (described below). Coral collections were done under a special license issued by the Guam Department of Agriculture–Division of Aquatic and Wildlife Resources to UOGML (in accordance with Section 63123 of Title 5, Guam Code Annotated). *Acropora abrotanoides* colonies were collected from Pago Bay and *Porites rus* colonies were collected from Western Shoals, Guam. Aside from being abundant in these collection sites [23], these species were selected because *A*. *abrotanoides* is among the most highly preferred prey coral species in Guam, whereas *P*. *rus* is one of the least preferred species [49]. Columnar and tabular/plate growth forms of these corals were collected to make surface area measurements easier. Coral infauna (e.g., *Trapezia* crabs) were physically removed from all coral fragments so as not to deter feeding by *A*. *planci* [50].

### Feeding Treatment

Nine female *A*. *planci* with intact arms and approximately similar diameter (ca. 320 ± 7 mm) were placed in individual plastic bins with flow through ambient seawater (temperature = 29.07 ± 0.47°C; salinity = 32.97 ± 0.06 psu; pH = 8.30 ± 0.05). All females were nearing reproductive maturity based on microscopic examination of oocytes drawn from starfish using the biopsy procedure described in the previous section. Oogenesis in *A*. *planci* usually takes between two and three months, although some oocytes can complete oogenesis within a month [51]. Starfish were assigned to one of three different feeding treatments (n = 3) for 60 days: i) Starved (no food), ii) *Acropora* (fed with *Acropora abrotanoides*), and iii) *Porites* (fed with *Porites rus*). Supply of coral food for fed treatments was replenished as soon as the piece of coral provided has been completely consumed. Since live coral was used in the diet of *ad libitum* fed starfish, the only way to reduce the amount of coral used was to minimize the sample size. Growth of each individual starfish was quantified based on changes in diameter (Δd) and weight (Δw) from day 0 to day 60. At the end of the feeding experiment (day 60) we also calculated the gonad index (GI) and the pyloric caeca index (PCI) for each individual. The average weight of gonads and pyloric caeca from three arms was multiplied with the total number of arms of each starfish to estimate the total gonad or pyloric caeca weight. GI and PCI were expressed as the ratio of gonad or pyloric caeca weight to the total weight of the starfish [8].

To relate differences in physiological and reproductive condition to food intake, we calculated the rate of feeding for each individual starfish by measuring the total surface area of coral consumed throughout the 60 day period. Consumed coral fragments were weighed dry and the surface area of each fragment was estimated following the foil-wrapping technique. Each piece of coral was tightly molded with heavy-duty aluminum foil to fit depressions and projections, following Marsh [52]. The aluminum foil molds were flattened and digital photographs were taken using a ruler as scale. These pictures were analyzed by calculating the area (cm^2^) of the flattened molds using the image analysis software, Image J [53].

### Spawning Induction and Oocyte Dimensions

To test the effect of feeding treatments on oocyte metrics, gonads were dissected from the nine females and ovaries were rinsed in 0.2-μm filtered seawater (FSW) to remove loose oocytes. Ovary lobes were treated in 10^−5^ M 1-methyladenine to induce ovulation. Released oocytes were transferred into containers with filtered seawater and wet mounted on glass slides for microscopic examination. Oocytes were photographed with a camera (Canon EOS 60D) mounted on a microscope (Leica DM300) with a calibrated ocular micrometer. Ayukai et al. [54] developed a criterion to estimate oocyte quality in *A*. *planci* based on morphometric characteristics. Oocytes that were relatively large (> 0.15 mm in diameter), spherical (round), and uniform in size and shape often achieved successful fertilization, embryogenesis, and gastrulation [54]. Diameters (d_oocyte_) of the long and short axes of 100 randomly selected mature oocytes (have undergone germinal vesicle breakdown) from each treatment were measured using Image J [53]. Oocyte volume (v_oocyte_) was calculated using the formula for an oblate spheroid: 4/3 × π × (long axis radius)^2^ × short axis radius. Oocyte sphericity is the ratio of the long and short axis diameter measurements.

### Fertilization

Oocytes from each female were placed in separate 1 L beakers with FSW kept at 28°C. Approximately 200 oocytes from each female were transferred into triplicate 250-ml beakers using a glass pipette. Spermatozoa were collected from the testes of 5 males and checked for motility under a microscope. Approximately equal amounts of spermatozoa from each male were combined and counted using a haemocytometer. Oocytes were fertilized with spermatozoa diluted to achieve a spermatozoa-to-oocyte ratio of 100:1. After 10 minutes, an aliquot of eggs was subsampled from each replicate and further development halted with 7% formalin before examination. One hundred eggs were examined and eggs with raised fertilization envelopes were counted and percent fertilization was calculated.

### Larval Rearing

**Fig 1:**
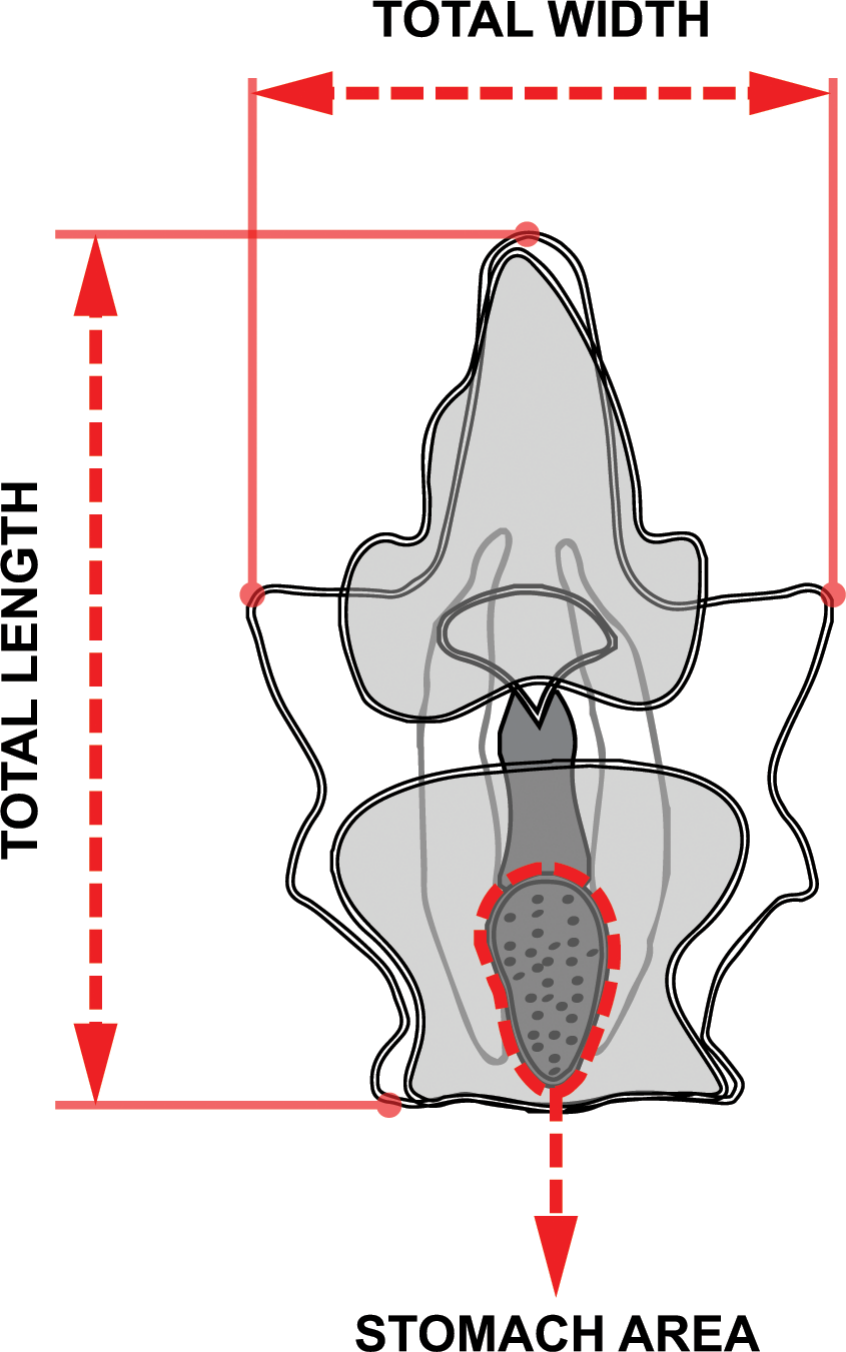
Bipinnaria larva morphometrics. Image analysis measurements of length, width, and stomach area of four-day old larvae.

Fertilized eggs from the nine females were separately reared in triplicates at 28°C and after 24 hours, 50 actively swimming gastrulae were siphoned into separate glass culture jars with 200 ml FSW. Each jar was equipped with a plastic stirring paddle attached to a 20-rpm synchronous motor. Water changes with fresh FSW were performed three times daily. Surviving larvae in each jar, regardless of developmental stage, were counted daily for eight days during the second water change. After four days, 10 normally developing larvae from each jar were placed in a relaxing agent (7% MgCl_2_) for 10 minutes and fixed in 10% formalin in FSW. Larvae were immediately photographed using a camera mounted on a microscope shortly after fixation and total length, width, and stomach area were measured using ImageJ (**Fig 1**).

After eight days, all surviving larvae were categorized into the following developmental stages: (1) early bipinnaria–preoral and anal lobes present, coelomic pouches below or close to mouth; (2) advanced bipinnaria–coelomic pouches above the mouth and almost touching, anterodorsal and posterolateral arms start to form; (3) late bipinnaria / early brachiolaria–anterior extension of fused coelomic sacs, anterodorsal and posterolateral arms longer, posterodorsal arms start to elongate, preoral arms start to form; and (–) abnormal (stunted, deformed) development [12,13,33,55]. The percentage of normally developing larvae after eight days was also calculated. No food was provided to the larvae during the entire duration of the experiment to control for variation in the food intake of individual larvae.

### Data Analyses

Mean daily consumption by each female was analyzed using one-way analysis of variance (ANOVA) to test for differences in total area of live coral consumed on exclusive diets of *Acropora* versus *Porites*. Change in diameter and weight (= post-treatment–pre-treatment), post-treatment gonad index, and post-treatment pyloric caeca index were compared between feeding treatments (3 levels, fixed) using one-way ANOVA followed by *post hoc* Tukey’s pairwise comparisons in SPSS 22.0 (IBM Corporation, NY, USA). Proportion of fertilized eggs were arcsine square-root transformed prior to nested ANOVA analysis with ‘Maternal Nutrition’ as fixed effects and ‘Female’ as a random factor nested under ‘Maternal Nutrition’. Normality and homogeneity of variance of data on oocyte dimensions, proportion of normal larvae, and proportion of larvae reaching late bipinnaria / early brachiolaria stage after eight days did not improve after transformations. A nested (hierarchical) permutational analysis of variance (PERMANOVA) with ‘Female’ (3 levels, random) nested within ‘Maternal Nutrition’ (3 levels, fixed) was run to analyze differences between and within feeding treatments. PERMANOVA is a non-parametric technique that may also be used in analyzing univariate data (Anderson et al. 2008). Analyses were conducted using the PERMANOVA+ add-on for PRIMER v.6 (Primer-E Ltd., Plymouth, UK), and used the Euclidean distance measure, Type III sums of squares, and 9999 permutations of the residuals under a reduced model to calculate the significance of the pseudo-F statistic. In cases where not enough unique permutations (< 100) were possible to determine permutational p-values (p_perm_), Monte-Carlo asymptotic p-values (p_MC_) were used instead [56]. Pairwise comparisons between Maternal Nutrition treatments were analyzed using Benjamini-Hochberg corrected p_MC_-values [57]. Data on the proportion of surviving larvae were arcsine square-root transformed prior to daily comparisons using nested ANOVA with a similar model described for fertilization data. Statistical comparison of larval length, width, and stomach area were made using a three-factor nested ANOVA with ‘Maternal Nutrition’ as a fixed effect (3 levels), ‘Female’ (3 levels, random) nested within ‘Maternal Nutrition’ and ‘Jar’ (3 levels, random) nested within ‘Female’ and ‘Maternal Nutrition’. A *post hoc* Tukey’s Test was used for pairwise comparisons of fixed factor means. Variation in the proportion of larvae under 4 larval development categories between feeding treatments was analyzed using G-test of independence followed by *post hoc* pairwise comparisons applying Benjamini-Hochberg correction for multiple comparisons [57].

## Results

### Coral Consumption and Morphometrics

Female *A*. *planci* provided with *ad libitum* rations of coral food exhibited a significant difference in consumption rates depending on the coral species (F_1,5_ = 55.309, p = 0.002). Starfish provided with *A*. *abrotanoides* consumed an average of 157.64 ± 10.71 cm^2^ of coral tissue per day while *P*. *rus* was consumed at rate of 101.81 ± 7.37 cm^2^ per day (**Table 1**). There was no significant difference in Δd between treatments (F_2,8_ = 3.413, p = 0.102); however, the change in weight (Δw) between treatments was significantly different (F_2,8_ = 5.816, p = 0.039) and *post hoc* pairwise comparisons show that weight gain in *Acropora*-fed females was significantly higher than in starved females (**Table 1**). Post-treatment gonad index (GI) was also significantly different between treatments (F_2,8_ = 7.530, p = 0.023), with values from *Acropora*-fed *A*. *planci* significantly higher than for starved starfish. Mean Δw and GI was not significantly different between *Acropora*- and *Porites*-fed starfish, nor between *Porites*-fed and starved females. Maternal treatments also had a significant effect on pyloric caeca index (PCI) values (F_2,8_ = 12.846, p = 0.007) and pairwise comparisons between treatments indicate that coral-fed starfish (regardless of whether they were maintained on *Acropora* or *Porites*) had higher pyloric caeca index values compared to starved *A*. *planci*. There was no significant difference in PCI between starfish in coral food treatments.

**Table 1:** Diameter and weight of females pre- and post-treatment, gonad index (GI) and pyloric caeca index (PCI) after feeding treatments.

Maternal Diet	Female	CC	d_0_	d_1_	Δd	w_0_	w_1_	Δw	GI	PCI
Starved	1	0	321	316	-5	1205	1154	-51	12.76	3.24
	2	0	330	327	-3	1022	997	-25	14.31	3.63
	3	0	314	313	-1	1069	1056	-13	15.15	5.26
*Acropora*	4	148	325	324	-1	1224	1219	-5	16.53	6.88
	5	155	311	314	3	1028	1046	18	18.22	8.66
	6	169	319	321	2	1154	1170	16	18.69	7.77
*Porites*	7	97	320	318	-2	1183	1172	-11	15.22	5.72
	8	98	313	316	3	964	971	7	16.02	6.97
	9	110	328	330	2	1257	1268	11	17.59	7.16

**CC** = mean daily coral consumption (cm^2^/day)

**d**_**0**_ = pre-treatment diameter (mm), **d**_**1**_ = post-treatment diameter (mm); **Δd** = diameter change

**w**_**0**_ = pre-treatment weight (g); **w**_**1**_ = post-treatment weight (g); **Δw** = weight change

**% GI** = post-treatment gonad weight (g) / post-treatment body weight (g) × 100%

**% PCI** = post-treatment pyloric caeca weight (g) / post-treatment body weight (g) × 100%

### Oocyte Dimensions and Fertilization

**Fig 2:**
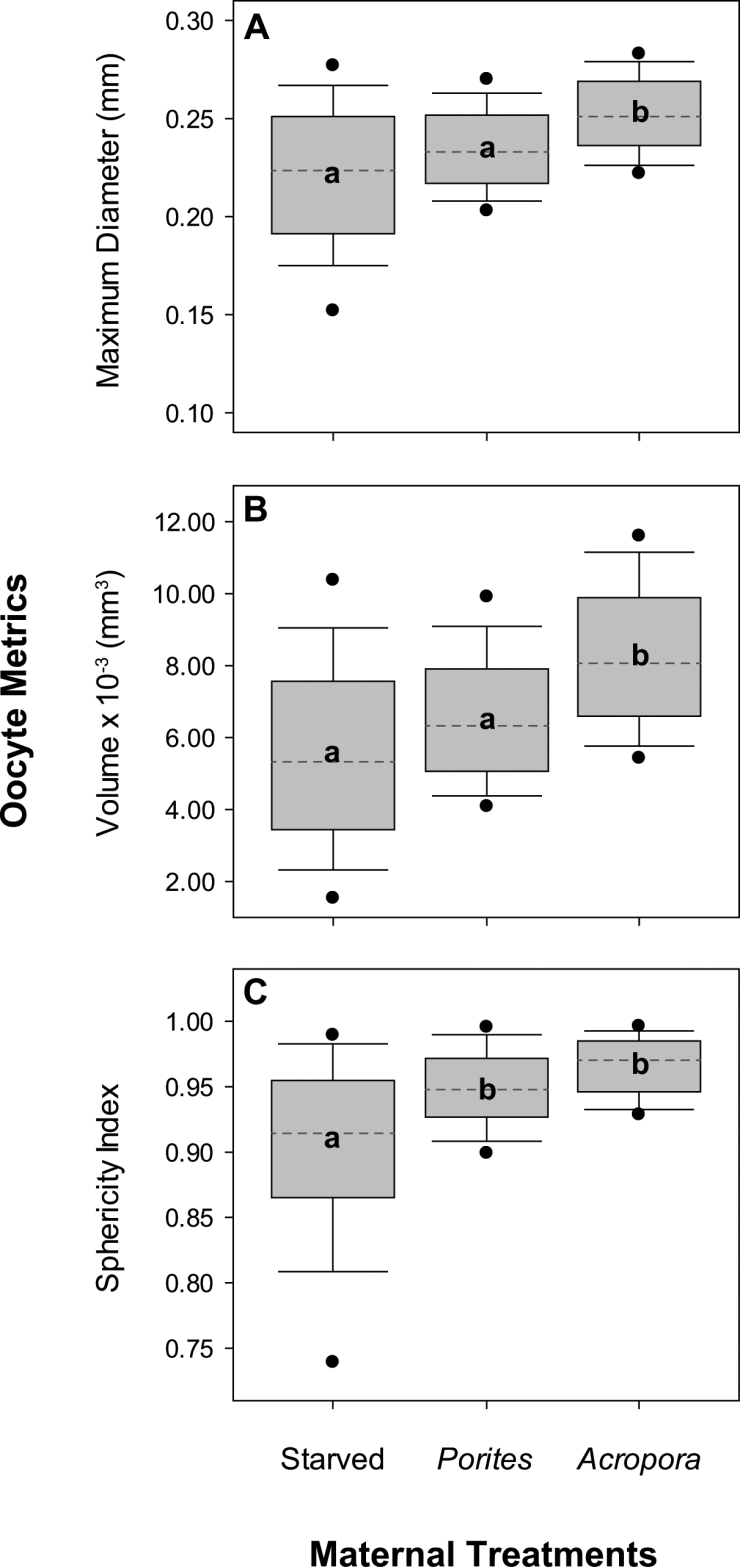
Size and shape of oocytes from females under different maternal nutrition treatments. Plots show median (dashed line), 25^th^ and 75^th^ percentile range in the grey box, 5^th^ and 95^th^ percentile range as error bars, and outliers as solid circles for oocyte (A) maximum diameter, (B) volume, and (C) sphericity index (n = 100). Different letters are significantly different based on *post hoc* pairwise comparisons.

Significant variation in oocyte diameter (pseudo-F_2,891_ = 11.463, p_perm_ = 0.009) and calculated oocyte volume (pseudo-F_2,891_ = 15.316, p_perm_ = 0.007) was noted among starfish in each of the three feeding treatments. The fixed effects accounted for 31% and 33.5% of variation in oocyte diameter and oocyte volume, respectively, while around 50% of variation was due to differences at the replicate level. Pairwise comparisons (**Fig 2**) showed that the diameter and volume of oocytes from *Acropora*-fed females (d_oocyte_ = 0.25 ± 0.02 mm; V_oocyte_ = 8.27×10^−3^ ± 1.95×10^−3^ mm^3^) was significantly higher than oocytes from *Porites*-fed (d_oocyte_ = 0.23 ± 0.02 mm; V_oocyte_ = 6.54×10^−3^ ± 1.77×10^−3^ mm^3^) and starved females (d_oocyte_ = 0.22 ± 0.04 mm; V_oocyte_ = 5.52×10^−3^ ± 2.63×10^−3^ mm^3^). Oocyte diameter in this study was relatively higher compared to previously reported oocyte sizes of *A*. *planci* and other coral reef asteroids with planktotrophic mode of development (**Table 2**). Maternal nutrition also had a significant treatment effect on oocyte sphericity (pseudo-F_2,891_ = 9.675, p_perm_ = 0.012), which accounted for 33.7% of variation while 47.1% of variation was due to differences at the replicate level. Oocytes from fed females (*Acropora* = 0.97 ± 0.02; *Porites* = 0.95 ± 0.03) were predominantly more spherical (ratio of long and short axis ≈ 1) than oocytes from starved females (0.90 ± 0.07), which were mostly ellipsoidal in shape. Oocyte diameter (pseudo-F_2,891_ = 9.759, p_perm_ < 0.001), volume (pseudo-F_2,891_ = 8.625, p_perm_ < 0.001), and sphericity (pseudo-F_2,891_ = 17.709, p_perm_ < 0.001) also differed significantly among females within maternal nutrition treatments, but only accounted for less than 20% of the total variation in each parameter. Overall, the size and shape of oocytes was less uniform in starved starfish compared to oocytes from fed starfish.

**Table 2:** Reported oocyte size of *A*. *planci* and other coral reef asteroids (Order Valvatida) from different locations.

Species	Diameter^a^ (mm)	Mode^b^	Location	Reference
Acanthasteridae				
*Acanthaster planci*	0.200–0.260	P	Maitre Is., New Caledonia	[8]
	0.100	P	GBR, Australia	[58]
	0.175	P	GBR, Australia	[3]
	0.180	P	GBR, Australia	[59]
	0.200–0.205	P	GBR, Australia	[60]
	0.100	P	Java, Indonesia	[61]
	0.189–0.205	P	Palau, Micronesia	[62]
	0.190	P	Guam, Micronesia	[12]
	0.125–0.287	P	Guam, Micronesia	This study^c^
	0.191–0.278	P	Guam, Micronesia	This study^d^
	0.214–0.288	P	Guam, Micronesia	This study^e^
	0.190	P	Kushimoto, Japan	[63]
Goniasteridae				
*Fromia ghardaqana*	1.000	L	Red Sea	[64]
Ophidiasteridae				
*Gomophia egyptiaca*	0.650	L	Guam, Micronesia	[65]
*Linckia laevigata*	0.150	P	Guam, Micronesia	[12]
*Ophidiaster granifer*	0.600–0.650	L	Guam, Micronesia	[66]
Oreasteridae				
*Culcita novaeguineae*	0.184–0.198	P	Palau, Micronesia	[62]
	0.180		Guam, Micronesia	[12]
*Protoreaster nodosus*	0.201	P	Palau, Micronesia	[62]

^**a**^ Oocyte diameter

^**b**^ Developmental modes**: P** = planktotrophic, **L** = lecithotrophic

^**c**^ Starved (from 3 females, n = 100 oocytes per starfish)

^**d**^ *Porites*-fed (from 3 females, n = 100 oocytes per starfish)

^**e**^ *Acropora*-fed (from 3 females, n = 100 oocytes per starfish)

**Fig 3:**
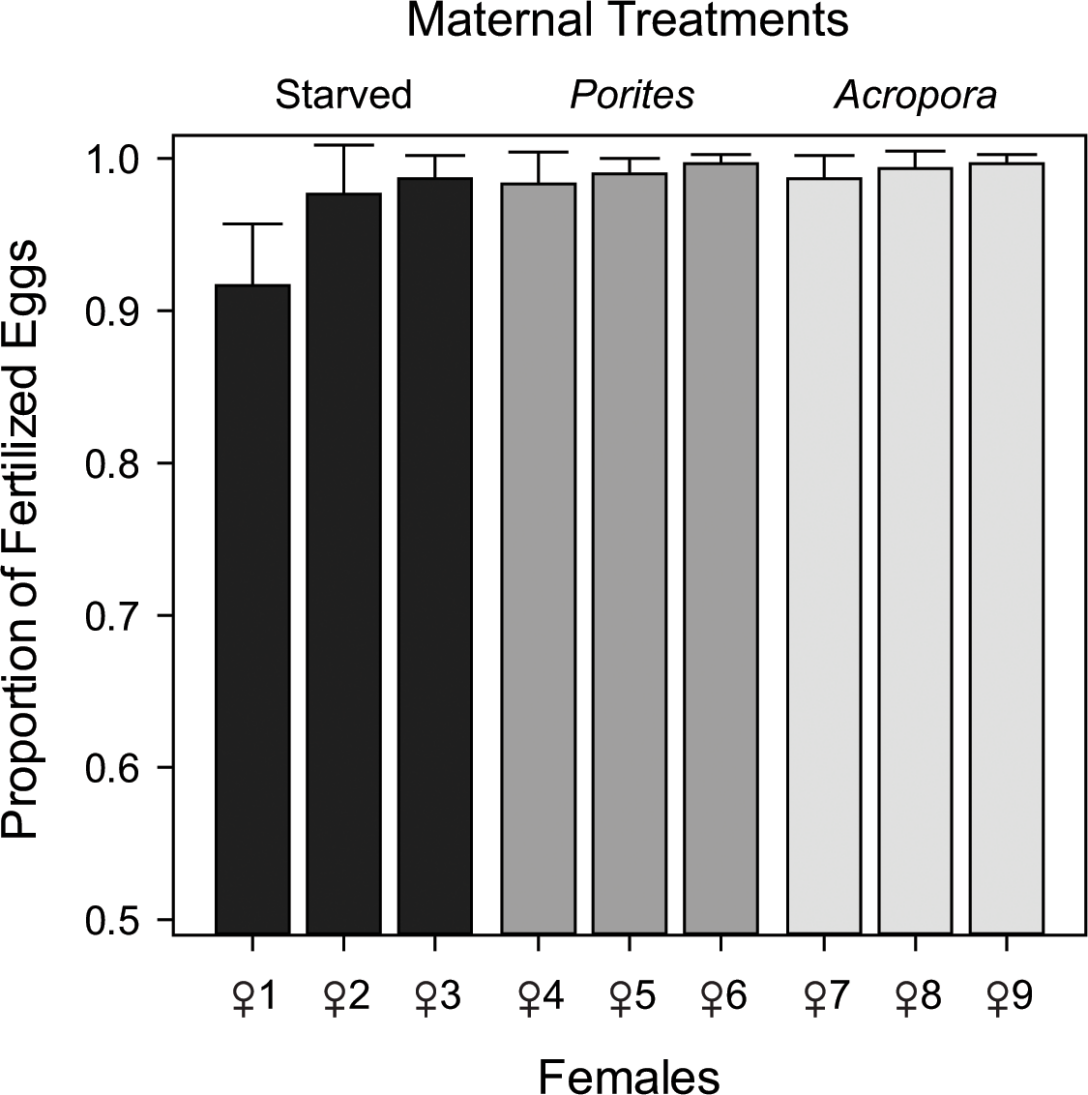
Fertilization success across all females under each maternal nutrition treatment. Proportion of fertilized eggs calculated from the number of eggs with raised fertilization envelopes out of 100 randomly selected eggs (n = 3). Error bars represent +1 standard deviation (SD).

Fertilization rates of eggs from individual females were high (88%-100%) across all treatment levels (**Fig 3**). Maternal nutrition did not have a significant effect on fertilization rates (F_2,18)_ = 2.318, p = 0.180), despite significantly different oocytes sizes. There was also no significant variation in fertilization rates of eggs from individual female starfish within treatments (F_2,18)_ = 2.477, p = 0.063).

### Larval Growth, Survival and Development

**Fig 4:**
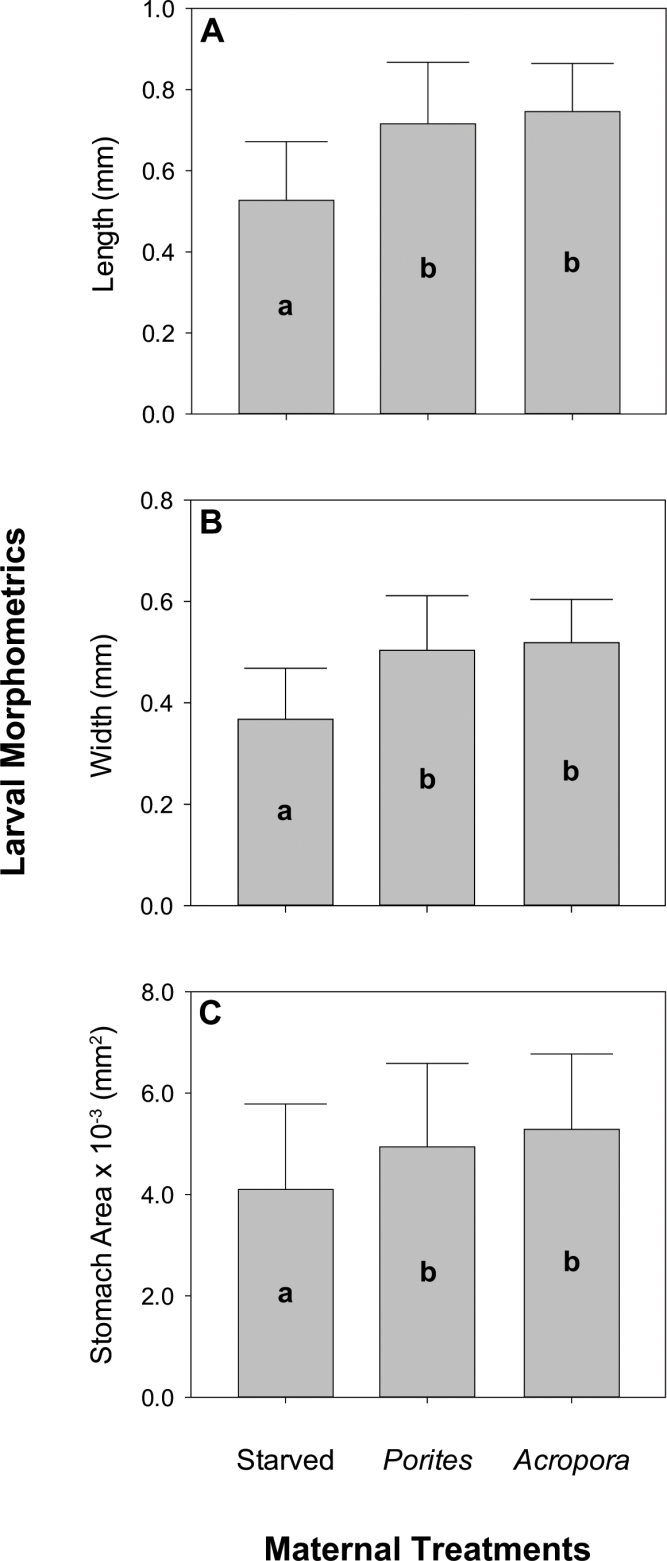
Morphometrics of larvae from females under different nutritional treatments. Image analysis measurements of (A) length, (B) width, and (C) stomach area (n = 10). Error bars are + 1 SD and different letters are significantly different based on Tukey’s *post hoc* test.

After four days, surviving larvae were in the bipinnaria stage. Maternal nutrition had a significant effect on larval morphometrics at this stage (**Fig 4**). Larvae from females fed with *Acropora* (0.75 ± 0.12 mm) and *Porites* corals (0.72 ± 0.15 mm) were significantly longer than larvae from starved females (0.53 ± 0.14 mm) (**Table 3**: F_2,243_ = 20.351, p = 0.002; **Fig 4A**). Maternal nutrition also had a significant effect on the width of larvae at this stage (**Table 3**: F_2,243_ = 23.321, p = 0.001, **Fig 4B**). Larvae from fed females (*Acropora*: 0.52 ± 0.09 mm; *Porites*: 0.50 ± 0.11 mm) were significantly wider than larvae from starved females (0.37 ± 0.10 mm). A similar pattern was also observed in terms of stomach area (**Table 3**: F_2,243_ = 23.321, p = 0.001, **Fig 4C**), where larvae from *Acropora*- (5.28×10^−3^ ± 1.49×10^−3^ mm^2^) and *Porites*-fed (4.94×10^−3^ ± 1.64×10^−3^ mm^2^) females had larger stomachs than larvae from starved females (4.10×10^−3^ ± 1.68×10^−3^ mm^2^). It is interesting to consider that 4 days may not have been sufficient time to discern larval growth trajectories between the two fed treatments.

**Table 3:** Results of mixed model hierarchical ANOVA for length, width, and stomach size of larvae from females under three treatments of maternal nutrition.

		Larval Length			Larval Width			Stomach Area		
Source	df	MS	F	p	MS	F	p	MS	F	p
Maternal Nutrition	2	1.267	20.351	0.002	0.624	23.321	0.001	33.405	32.564	0.001
Female(Maternal Nutrition)	6	0.062	2.128	0.100	0.027	2.000	0.119	1.026	0.522	0.784
Jar(Female(Maternal Nutrition))	18	0.029	1.666	0.046	0.013	1.491	0.094	1.964	0.736	0.772
Error	243	0.018			0.009			2.668		

**Fig 5:**
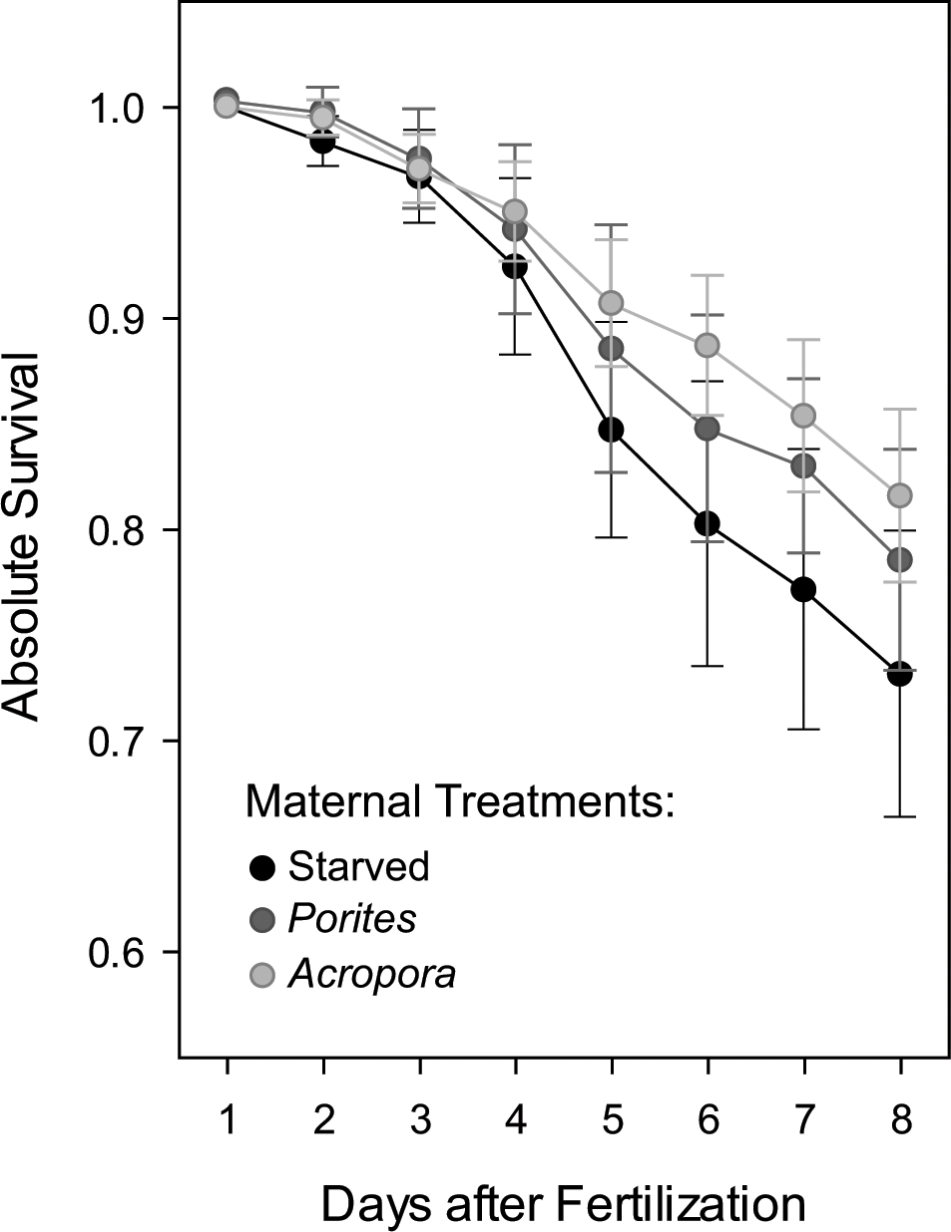
Daily survival rates of larvae reared for eight days. Data points are mean values ± 1SD of pooled proportions of surviving larvae from all females and rearing jars under each maternal nutrition treatment (n = 9).

**Fig 6:**
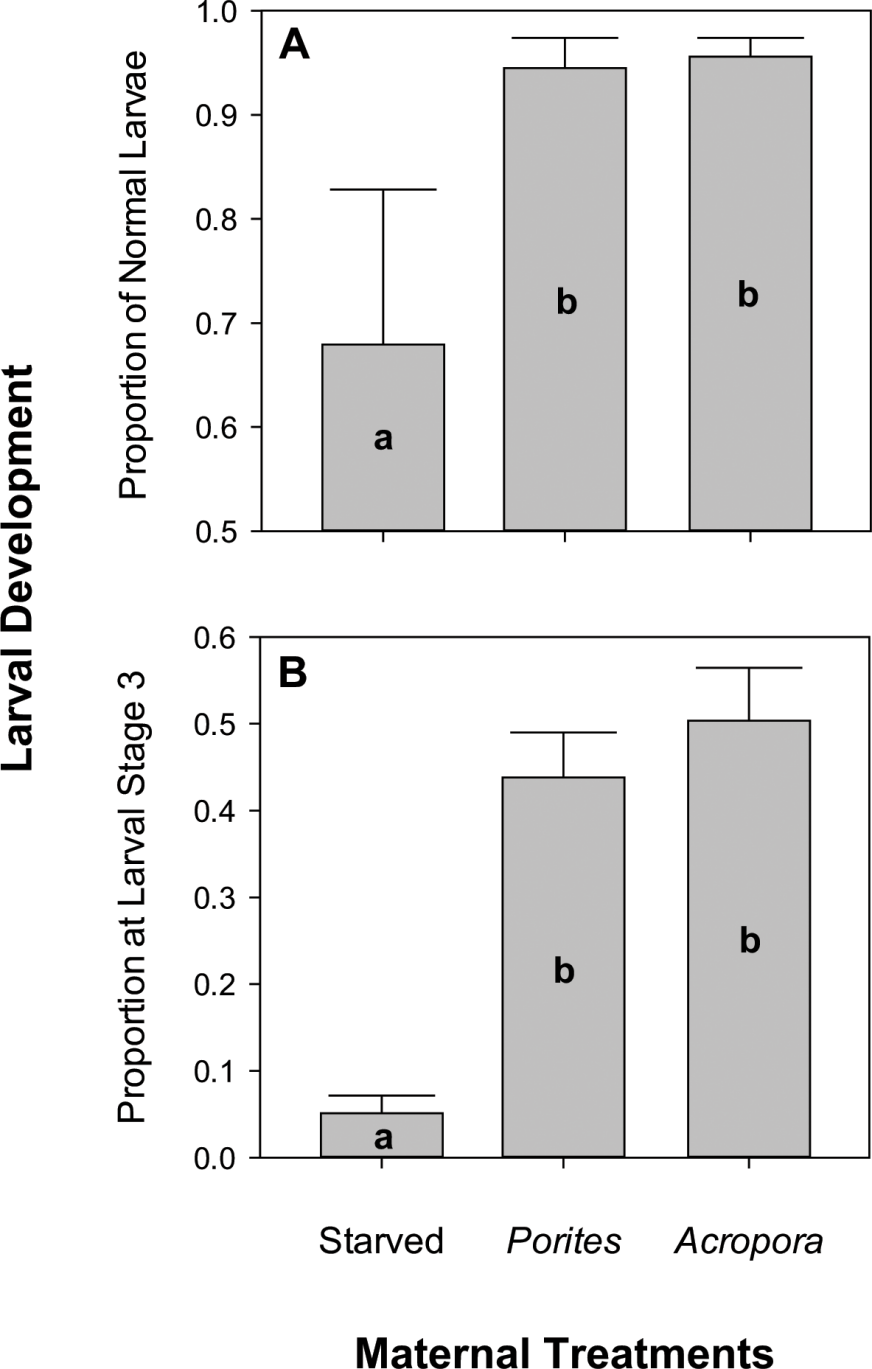
**Proportion of (A) normal larvae and (B) late bipinnaria / early brachiolaria larvae at day eight.** Error bars represent + 1SD and n = 9 for each maternal treatment. Different letters are significantly different based on Tukey’s *post hoc* tests.

**Fig 7:**
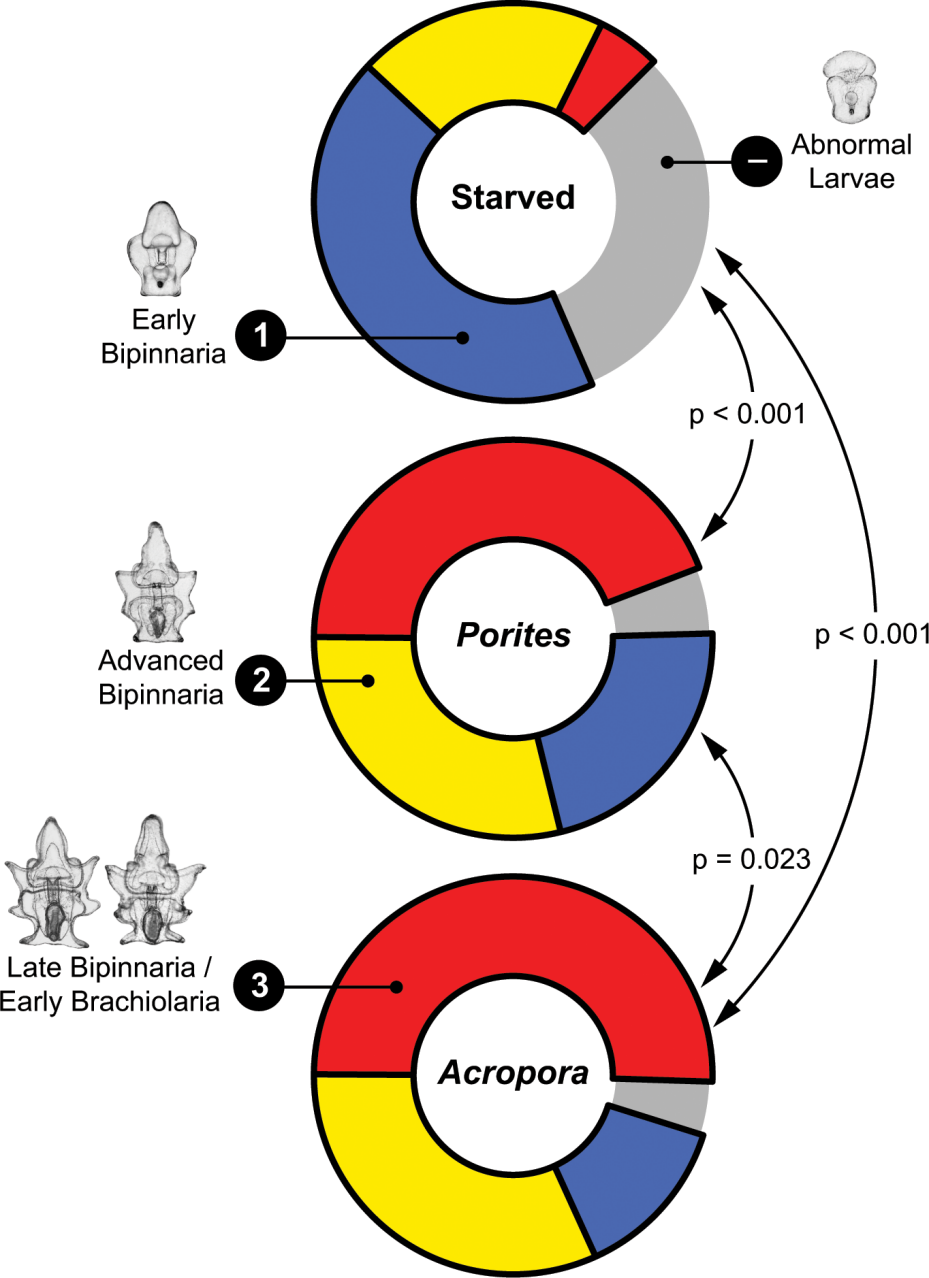
**Proportion of larvae under 4 development categories: (1) early bipinnaria, (2) advanced bipinnaria, (3) late bipinnaria / early brachiolaria, and (–) abnormal larvae.** Arrows and p-values represent *post hoc* G-test pairwise comparisons with Benjamini-Hochberg-corrected significance levels.

Maternal nutrition had no significant effect on daily survival rates (**Fig 5**), which remained above 60% across all levels after eight days of rearing. The highest mortality across all treatments was recorded four days after fertilization; starved females, in particular, decreased by an average of 8% at day four. Conversely, maternal nutrition had a significant effect on the proportion of larvae that developed normally (pseudo-F_2,18_ = 8.192, p_perm_ = 0.011; **Fig 6A**). Among the surviving larvae after eight days, there was a higher proportion of normally developing larvae in the *Acropora-* (96 ± 2%) and *Porites*-fed (94 ± 3%) treatments compared to starved (68 ± 15%) treatments. This pattern was even more apparent when the proportion of larvae that reached the late bipinnaria / early brachiolaria stage was compared (pseudo-F_2,18_ = 177.720, p_perm_ = 0.004; **Fig 6B**)**.** The proportion that reached the late bipinnaria / early brachiolaria stage was ten times higher in the *Acropora* treatment (50 ± 6%) and nine times higher in the *Porites* treatment (44 ± 5%) compared to the starved treatment (5 ± 2%). Overall, the proportion of larvae that progressed to a new developmental stage was significantly different between treatments (G = 339.555, df = 6, p < 0.001; **Fig 7**). All pairwise comparisons were significant at Benjamini-Hochberg corrected alpha levels (**Fig 7**). There was a higher proportion of abnormal (31%) and early bipinnaria (43%) larvae in the starved treatment compared to the fed treatments. Larvae under the *Acropora* treatment were in relatively advanced stages with 32% at advanced bipinnaria and 50% at late bipinnaria / early brachiolaria stage. All normal larval stages were represented in the *Porites* treatment consisting of 22% at early bipinnaria, 29% at advanced bipinnaria, and 44% at late bipinnaria / early brachiolaria stage.

## Discussion

Average daily coral consumption *of A*. *planci* in this study falls within feeding rates observed in the field during summer months on the Great Barrier Reef [67]. Consistent with field observations [24,25], consumption rates on *A*. *abrotanoides* was significantly higher compared to feeding rates on *P*. *rus*. One possible explanation for marked feeding preferences among corallivorous organisms (given that the order of preferences is not always consistent with energetic value of the different corals, e.g., Keesing [68]) is that preferred corals are those upon which feeding is most efficient, e.g. Cole and Pratchett [69]. Accordingly, we found that the consumption rates of *A*. *planci* on *A*. *abrotanoides* (based on estimated area of live coral consumed each day) were 50% higher compared to similar sized starfish feeding on *P*. *rus*. This is because *A*. *planci* can digest the tissues of *Acropora* much more efficiently than tissues of *Porites* [68]. Moreover, *Acropora* corals tend to have much deeper tissue layers than *Porites*, owing to their perforate skeleton [70]. Given that *Acropora* corals have higher energetic content and greater tissue depth compared to *Porites*, and can also feed at greater rates on *Acropora*, it is expected that *A*. *planci* on *Acropora* would be in much better nutritional condition.

Despite differences in diet and food intake, no differences were apparent in the physical appearance of female *A*. *planci* that were starved for 60 days versus individuals maintained on exclusive diets of *A*. *abrotanoides* or *P*. *rus*. This reflects the considerable resilience of starfish to shortages in prey [71]. However, differences in prey intake did appear to have a significant effect on the physiological condition of crown-of-thorns starfish, with flow-on effects for individual reproductive capacity. Most notably, the starfish that were fed (even if on sub-optimal coral prey, *P*. *rus*) tended to increase in weight over the course of the study, whereas starved individuals consistently lost weight. Fed individuals also had heavier gonads and pyloric caeca compared to starved individuals, even when standardized for overall body weight, as shown for other echinoderms [21]. Xu and Barker [35] suggest that the pyloric caeca act as nutrient reservoirs to support reproductive and maintenance activities under conditions of nutritional stress.

Increases in gonad weight of well-fed echinoderms can be attributed to increased oocyte size and/or higher maternal fecundity [72]. Similarly, the starfish, *L*. *epichlora*, had higher fecundity and produced bigger oocytes at sites with increased food availability [43]. Bertram and Strathmann [32] found that gonad volume increased as a function of oocyte size in *S*. *droebachiensis*. The weight of *A*.*planci* gonads also partly reflects increased fecundity, whereby Conand [8] estimated that *A*.*planci* consistently produce 90,190 oocytes per gram of ovary. Our study clearly shows that individual starfish feeding on *A*. *abrotanoides* had proportionally larger gonads, and produced larger oocytes (diameter and volume) compared to *Porites*-fed and starved females. Oocytes from *A*. *planci* that were fed with *A*. *abrotanoides* were also more uniform and more spherical in shape compared to oocytes from starved females, which is generally reflective of higher oocyte quality, as well as leading to increased rates of fertilization and larval development [54].

It is also noteworthy that average oocyte diameter measurements in this study were relatively bigger than previously reported measurements for *A*. *planci* (**Table 2**). Since the mean oocyte diameter of starved treatments (0.22 ± 0.04 mm) in this study was still marginally higher than measurements from other localities, this variation may largely be from the source population and only partially due to the experimental manipulation of diet. More importantly, the biochemical and energetic composition of oocytes warrant further investigation because these measurements show that oocytes of *A*. *planci* are relatively bigger compared to oocytes from echinoderms that are obligate planktotrophs [73]. Yolk-rich planktotrophic larvae of the Antarctic starfish, *Porania antarctica* have been found to differentiate to brachiolaria stage and increase in length even in the absence particulate food [74]. This may help explain extreme population fluctuations in *A*. *planci* compared to other asteroids with similar planktonic life histories.

For *A*. *planci*, fertilization rates were not significantly different among treatments and were consistently high across all females regardless of differences in nutritional conditions, oocyte size and shape. Similarly, no significant variation in fertilization success was observed when the sea urchin, *S*. *droebachiensis*, was fed with artificial diets containing different levels of dietary protein and additives [41]. However, Levitan [42] has shown that for the sea urchin *Strongylocentrotus franciscanus*, fertilization rates were higher on larger eggs, but only under sperm-limiting conditions. Variation in oocyte shape may also result in constrained or arrested development. For example, experiments in which echinoderm oocytes were artificially deformed resulted in abnormal cleavage patterns [75]. Conversely, Podolsky and Strathmann [76] suggested that varying oocyte shape could provide a mechanism to increase oocyte-spermatozoa collisions without increasing oocyte volume. Our results warrant further investigation into whether any oocyte size and shape has an effect on fertilization rates at lower spermatozoa concentrations and whether the effect of environmental conditions could swamp the influence of gamete traits. High spermatozoa concentrations were used in this study to evaluate whether low maternal investment reduces fertilization rates. Spawning in turbulent water conditions, lack of synchrony, and low proximity to other spawning individuals could potentially limit spermatozoa concentrations and oocyte traits may be under intense selection to increase fertilization rates in the field.

Females that were fed (regardless of whether they were fed with *A*. *abrotanoides* or *P*. *rus*) produced larger larvae with larger stomachs compared to starved females. Maternally derived energetic lipids, particularly triglycerides, fuel larval development in planktotrophic starfish [47]. High feeding rates by *A*. *planci* on a lipid-rich food source, such as corals [77,78], may allow excess resources for gametogenesis and provide increased maternal provisioning of lipids as a buffer against unfavorable nutritional conditions during the planktonic larval stage [45]. For instance, differences in oocyte triglyceride levels were still observable at the bipinnaria stage for the planktotrophic starfish, *Meridiastra mortenseni*, indicating flow-on effects for larval fitness [47]. The same major energetic lipid class (i.e. triglycerides) sequestered by feeding echinoderm larvae to support early juveniles is also provided by the female parent to fuel early development [79]. Hence, additional energetic reserves allow larvae to withstand prolonged periods of starvation [39] and also may be used to produce larger larvae with morphologies that improve feeding effectiveness [60]. These early larval stage metrics are useful indicators since larger larvae usually progress much faster to advanced stages and have higher survival later in development even at unfavorable food conditions for larvae [33]. Although exogenous food supply may still be necessary to complete metamorphosis, faster growth reduces planktonic larval duration and exposure to larval predators [48].

Overall, survival rates were high across all treatments (>60%). Abnormal larvae continue swimming in the water column and remain alive for extended periods, but do not develop further [5]. The proportion of stunted or deformed larvae from starved females was significantly higher and most larvae under this treatment remained at the early bipinarria stage after eight days. Even at starved larval conditions, as is the case in this study, a large proportion of larvae from fed females progressed from early bipinnaria to late bipinnaria / early brachiolaria stage. This implies that any surplus of maternally derived energetic lipids may support development even at conditions when exogenous food resources are limited. In assessing the role of exogenous food availability on larvae from these different conditions of maternal diet, the patchy nature of planktonic food resources must be considered [80]. At low phytoplankton levels, developing larvae of *A*. *planci* may also exploit other sources of food to supplement endogenous nutrient reserves [16]. When planktonic food is abundant, larvae of *S*. *droebachiensis* adults from nutritionally rich habitats have been shown to metamorphose sooner than larvae from adults collected from habitats with low food availability [81].

In summary, *A*. *planci* given almost limitless access to *A*. *abrotanoides*, which is among the most preferred coral prey, increased in weight and had heavier gonads compared to starved females. *Acropora*-fed females also produced larger oocytes compared to *Porites*-fed and starved females. Fed starfish produced bigger larvae with larger stomachs and had a higher frequency of normally developing larvae. Females on *Acropora* diet also produced larvae that progressed to more advanced stages faster compared to *Porites*-fed starfish, which progressed faster than starved starfish. These results show that the influence of maternal diet on oocyte characteristics was carried over to early larval stages, affecting both larval size and development. Variability at these earlier stages of development has been known to persist even after metamorphosis and impact juvenile quality [43,82]. This has significant implications for the reproductive capacity of female starfish living in reef habitats with varying coral community structure and abundance. Importantly, the local abundance of preferred prey (e.g., *Acropora*) could have an important impact on the overall reproductive capacity and resistance to larval starvation in the absence of phytoplankton blooms. Dense aggregations of brachiolaria-stage larvae of *A*. *planci* were detected in areas where phytoplankton concentrations were low [83], suggesting that apart from larval nutrition provided by nutrient-rich waters, other factors may play a role in larval survival and development. Future studies need to carefully consider the nutritional condition of females from which oocytes are collected in looking at the effect of larval nutrition on development. Future work should also measure the biochemical composition of oocytes from females under different nutritional states and also contrast the magnitude of the effects of maternal nutrition (endogenous) and larval nutrition (exogenous) on larval vitality and morphometry to see if these differences disappear through compensation or persist throughout development.
